# Viral Infection Modulates Mitochondrial Function

**DOI:** 10.3390/ijms22084260

**Published:** 2021-04-20

**Authors:** Xiaowen Li, Keke Wu, Sen Zeng, Feifan Zhao, Jindai Fan, Zhaoyao Li, Lin Yi, Hongxing Ding, Mingqiu Zhao, Shuangqi Fan, Jinding Chen

**Affiliations:** 1 College of Veterinary Medicine, South China Agricultural University, No. 483 Wushan Road, Tianhe District, Guangzhou 510642, China; 18306616234@163.com (X.L.); 13660662837@163.com (K.W.); senzeng@stu.scau.edu.cn (S.Z.); zhaofeifan1234@163.com (F.Z.); fanjindai@stu.scau.edu.cn (J.F.); lizhaoyao@stu.scau.edu.cn (Z.L.); yilin@scau.edu.cn (L.Y.); dinghx@scau.edu.cn (H.D.); zmingqiu@scau.edu.cn (M.Z.); 2 Guangdong Laboratory for Lingnan Modern Agriculture, College of Veterinary Medicine, South China Agricultural University, Guangzhou 510642, China; 3 Key Laboratory of Zoonosis Prevention and Control of Guangdong Province, Guangzhou 510642, China

**Keywords:** mitochondrial fission and fusion, virus infection, apoptosis, host innate immunity

## Abstract

Mitochondria are important organelles involved in metabolism and programmed cell death in eukaryotic cells. In addition, mitochondria are also closely related to the innate immunity of host cells against viruses. The abnormality of mitochondrial morphology and function might lead to a variety of diseases. A large number of studies have found that a variety of viral infections could change mitochondrial dynamics, mediate mitochondria-induced cell death, and alter the mitochondrial metabolic status and cellular innate immune response to maintain intracellular survival. Meanwhile, mitochondria can also play an antiviral role during viral infection, thereby protecting the host. Therefore, mitochondria play an important role in the interaction between the host and the virus. Herein, we summarize how viral infections affect microbial pathogenesis by altering mitochondrial morphology and function and how viruses escape the host immune response.

## 1. Physiological Morphology of Mitochondria

Mitochondria originated from an ancient bacterial endosymbiont, are important organelles found in almost all cells. In the nearly 130 years since mitochondria were first reported, new functions have been discovered. Mitochondria maintain the dynamic balance of the mitochondrial network through the fission and fusion mediated by a dedicated set of dynamin-related GTPases, provide energy for cells, and regulate processes such as autophagy, calcium homeostasis, innate immunity, signal transduction, and apoptosis [1].

Mitochondria are in a highly dynamic process within the cell, undergoing fission and fusion cycles to control the mitochondria’s morphology. The Fuzzy onion (Fzo) is the first protein discovered to mediate mitochondrial fusion during Drosophila spermatogenesis, and mutations in the Fzo gene can cause mitochondrial fusion disorders and abnormal accumulation in Drosophila sperm cells [2]. In mammals, the proteins that mediate mitochondrial fusion mainly include Mfn1 (Mitofusin1), Mfn2 (Mitofusin2), and OPA1 (Optic atrophy 1) [3,4,5]. Mfns contain heptad repeat regions (HR2), and Mfn1 and Mfn2, located on the outer mitochondrial membrane (OMM), interact to form Mfn1/Mfn2 homodimers or Mfn1/Mfn2 heterodimers through oligomerization of the HR2 structures, thereby promoting phase trans-plugging of the adjacent OMM [6,7], and involving GTP hydrolysis, which eventually leads to the fusion of the OMM [8,9]. OPA1, a dynamically related GTPase and localized to IMM, participate in IMM fusion. OPA1 protein was hydrolyzed into different fragments in the intermembrane space: one is the long subtype L-OPA1 associated with mitochondrial fusion, the other is the short subtype S-OPA1 [10,11]. L-OPA1 achieves selective mitochondrial fusion through the heteromorphic interaction between its GTPase domain and its adjacent mitochondrial membrane cardiolipid (CL). Loss of fusion-mediating proteins (MFN1, MFN2, and OPA1) can cause changes in mitochondrial morphology, leading to mitochondrial fragmentation. Mitochondrial fusion is a necessary cellular process that facilitates the merging of mitochondrial fragments and mediates the exchange of mitochondrial DNA, proteins, and metabolites. 

Mitochondrial consonance proteins were deleted by gene knockout and RNA interference techniques, leading to mitochondrial fragmentation [3,5]. Mitochondria can also break down damaged mitochondria through “mitochondrial fission”, breaking them down into smaller fragments. In mammalian cells, Drp1 is a significant protein that mediates mitochondrial fission. After Drp1 is activated, it is recruited from the cytosol to the OMM, where oligomerization occurs. Drp1 forms rings and spirals within the diameter of the OMM and hydrolyzes GTP depending on its GTP-enzyme activity, resulting in membrane constriction and scission [12,13]. The transport and function of Drp1 are rapidly regulated by the opposing effects of phosphorylation at two key serines. In general, phosphorylation of serine 616 enhances Drp1 activity and promotes its targeting of mitochondrial aggregation, while phosphorylation at serine 637 reduces the activity of Drp1, keeping it in the cytoplasm [14]. For example, RIP1 phosphorylates Ser616 residue of Drp1, thereby inducing mitochondrial fission and eliminating damaged mitochondria through mitophagy, when cells are in a state of energy stress [15]. Phosphorylation at Ser637 of Drp1 inhibits the interaction of GTP-binding/middle domains with the GED domain, thereby reducing GTPase activity and altering Drp1 function and mitochondrial morphology [16]. Drp1 requires proteins of various accessories to perform its function. At present, mitochondrial fission factor (mitochondrial fission factor, Mff), mitochondrial fission protein 1 (mitochondrial fission protein 1, Fis1), mitochondrial dynamin 49 (mitochondrial dynamics proteins of 49 kDa, Mi D49), and mitochondrial dynamin 51 (mitochondrial dynamics proteins of 51 kDa, MiD51) located on the mitochondria have been found to act as ligands for Drp1, which recruit Drp1 to the mitochondria and regulate mitochondrial fission [17]. Fis1, the only Dnm1 receptor in yeast cells, is controversial for recruiting Drp1 to mitochondria in mammalian cells. For example, Fis1 and Drp1 interact in mammalian cells, and increasing Fis1 levels will promote mitochondrial fission [18]. However, Fis1 deletion in colon cancer cells suggests that it is not necessary for mitochondrial division [19]. A recent study found that human Fis1 blocks mitochondrial fusion mechanisms by binding to Mfn1, Mfn2, and OPA1, suggesting that Drp1 is dispensable for human Fis1 function [20]. Mff protein is also a receptor molecule of Drp1, it interacts with Drp1 through the amino-terminal cytoplasmic region, and is distributed homogeneously on the OMM, mainly in the same places as Drp1 [19]. Overexpression of Mff can promote the recruitment of Drp1 to mitochondria, while silencing of Mff expression can promote mitochondrial fusion. In addition, mitochondrial dynamics proteins (MiDs) are involved in mitochondrial fission in fis1 and Drp1 deficient cells. When MiDs are overexpressed, they recruit a large number of inactive S637 phosphorylated Drp1 into mitochondria to mediate mitochondrial elongation [21,22].

Mitochondria are involved in a series of cellular activities such as cell metabolism, programmed cell death and innate immunity, and the host response to viral infection. In addition, in the long-term evolutionary process, viruses have evolved a pathway to affect their intracellular survival by targeting mitochondria, and by mediating mitochondria-induced cell death, they can spread or evade host immunity. In this review, we explore how viruses manipulate mitochondria and how this manipulation affects microbial pathogenesis.

## 2. Viral Infection Disrupts Mitochondrial Dynamics

A variety of viral infections can induce mitochondrial autophagy by destroying the dynamic balance of mitochondria, which is conducive to viral self-infection. Since the early discovery of mitochondrial morphological changes in patients with Hepatitis C virus (HCV), more and more studies have focused on the changes in mitochondrial function caused by HCV infection, which is a positive-strand RNA virus [23]. The HCV core protein can be targeted and located on the OMM, resulting in a decrease in electron transport complex I, inhibition of mitochondrial electron transport, and an increase in the production of reactive oxygen species (ROS) [24,25]. HCV also induces ROS production through Core, E1, and NS3 proteins, which triggers mitochondrial permeability transition, leading to DNA damage and STAT3 activation [26]. Reducing the mitochondrial permeability threshold induced by Ca2+ and ROS is a feature of hepatitis C virus infection. It is a direct result of the interaction of HCV core proteins with mitochondria [27]. HCV infection also disrupts mitochondrial dynamics by promoting mitochondrial fission and mitophagy to promote viral persistence. HCV induces the phosphorylation of Drp1 (Ser616) and transports it to the mitochondria to mediate mitochondrial fission, thereby causing mitophagy [28]. Interference of HCV-induced mitochondrial fission and mitophagy can reduce glycolysis and ATP production as well as increase interferon synthesis, thereby inhibiting viral secretion [28]. Another study showed that HCV-induced mitochondrial fission is not only dependent on DRP1 protein, but HCV NS5A protein can also interact with phosphatidylinositol 4-kinase IIIα, which induces mitochondrial fragmentation [29]. HCV induces the expression of Parkin and PINK1, and triggers the translocation of Parkin into mitochondria to mediate mitophagy. Inhibition of mitophagy by silencing Parkin and PINK1 can partially rescue mitochondrial complex I enzyme activity and inhibit HCV replication [28]. Interestingly, the HCV core protein interacts with Parkin, inhibiting Parkin translocation to mitochondria, leading to the formation of mitochondrial autophagosomes and the failure of autophagy degradation [30]. Classical swine fever virus (CSFV) and Dengue virus (DENV) belong to the same family of flaviviruses as HCV does, and infection can also facilitate self-replication by affecting mitochondria’ function [31,32,33,34,35]. CSFV infection causes MNF2 to be ubiquitinated and degraded and stimulates the expression of Parkin and PINK1 and mitochondrial translocation, leading to mitochondrial fission and increased mitophagy. Silencing DRP1 and Parkin resulted in a decline in CSFV replication [31]. DENV proteins NS4B and NS3 mediate an imbalance in mitochondrial dynamics by inhibiting Drp1-triggered mitochondrial fission, which is conducive to the replication of DENV. In addition, NS4B protein of DENV can inactivate DRP1 and mediate mitochondrial elongation [34]. Mitochondrial extension brings mitochondria into contact with convoluted membranes (CMs) and destroys the integrity of the mitochondria-endoplasmic reticulum binding site on the mitochondrial-associated membrane (MAM), resulting in RLR signal transduction failure and reduced interferon production. However, another study found that DENV can also inhibit mitochondrial fusion through NS2B3 protein cleavage of MFN1 and MFN2, blocking RLR signal transduction and destroying mitochondrial membrane potential, thereby enhancing DENV infection [35].

**Figure 1:**
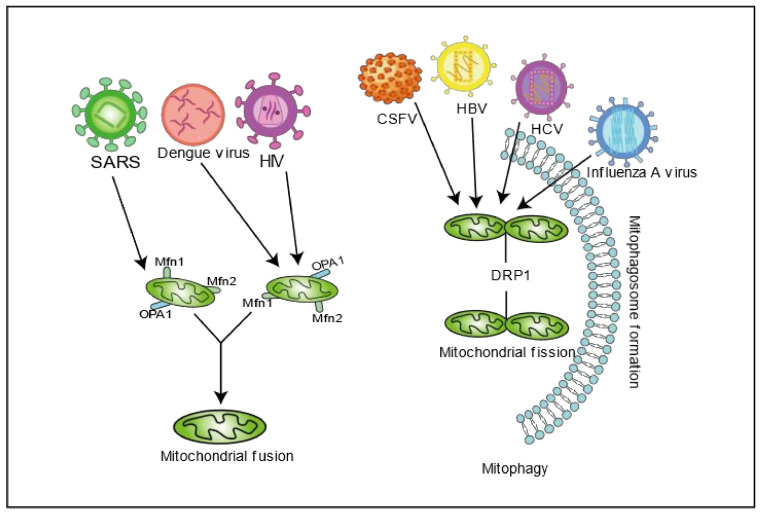
Viral infection disrupts mitochondrial dynamics. Different viruses affect mitochondrial dynamics through mitochondrial fusion proteins (MFNs, OPA1) or fission proteins (DRP1) and induce mitophagy to clear damaged mitochondria to enhance cell survival and viral persistence.

It has also been reported that hepatitis B virus (HBV), a partially double-stranded DNA virus belonging to the *Hepatoviridae* family, may mediate mitochondrial damage in liver cells by changing mitochondrial dynamics, thus causing liver diseases. Many studies have reported that HBV HBx protein can target mitochondria and be situated in the OMM, IMM, or matrix. Studies have shown that MARCH5, a mitochondrial E3 ubiquitin ligase, can degrade HBx accumulated on mitochondria through polyubiquitination and regulate mitochondrial dynamics through ubiquitination of Drp1, Fis1, and Mfn1, thereby negatively regulating HBV [36]. HBx recruits Parkin to destroy depolarized/dysfunctional mitochondria by up-regulating PINK1 expression [37]. Other studies have shown that HBV and HBx protein promoted mitochondrial fission by promoting the expression of DRP1. HBV and HBx protein also promote cell survival and persistent viral infection via stimulating Parkin-mediated mitophagy [37]. PB1-F2 is a crucial virulence factor for the influenza virus’ pathogenicity, which is an enveloped RNA virus of the orthomyxoviridae family. PB1-F2 targets mitochondria and is transported to the IMM through the TOMM40 channel, causing loss of mitochondrial membrane potential and disrupting mitochondrial function [38,39,40]. In contrast, the low pathogenic subtype influenza A PB1-F2, lacking the c-terminal region, does not cause mitochondrial dysfunction [41]. PB1-F2 interacts with TUFM (Tu translation elongation factor, mitochondrial) on mitochondria and induces mitophagy and inhibits type I interferon expression [42]. However, a recent study showed that H1N1 infection can promote mitochondrial elongation and alter the host cell’s endoplasmic reticulum–mitochondrial contact sites by increasing OPA1 expression and decreasing DRP1 expression, thereby altering mitochondrial morphology dynamics. In addition, the treatment of cells with Mito-C (a novel pro-fission compound) significantly reduced viral replication by restoring part of the mitochondrial function [43]. Severe Acute Respiratory Syndrome Coronavirus (SARS-CoV) is a single-stranded positive-stranded RNA virus belonging to the genus Coronavirus. Its NSP2 interacts with PHB1 and PHB2, implicated in several cellular functions, thereby impairing intracellular signaling and affecting mitochondrial biogenesis [44,45]. SARS-CoV virulence factor ORF-9B also degrades DRP1 through the proteasome, leading to mitochondrial fusion that evades the host innate immune response [46]. Severe Acute Respiratory Syndrome Coronavirus 2 (SARS-CoV-2), a member of the same family as SARS-CoV, has caused global social and economic disruption. Recent studies have shown that SARS-CoV-2 could manipulate immune response and cell metabolism to promote cell replication by regulating autophagy, increasing ROS processes, and decreasing mitochondrial function [47]. In SARS-CoV-2, ORF9b interacts with the TOM70 subunit of the OMM protein import mechanism [48], which has a potential regulatory effect on MAVS. SARS-CoV-2 Nsp4, required for CM formation in SARS-CoV, potentially interacts with the mitochondrial import machinery (TIM) complexes [48]. SARS-CoV-2 Nsp8 also interacts with mitochondrial ribosomes [48]. More and more studies have shown that viruses maintain the viral replication’s ecological sites by manipulating mitochondrial dynamics (Figure 1). Therefore, the study of virus and mitochondrial dynamics may become the critical drug targets for viral infection treatment.

## 3. Viral Infection Regulates Mitochondria-Induced Cell Death

Apoptosis is the process of cell autonomy and programmed death, controlled by genes, to maintain the stability of the internal environment. At present, cell apoptosis can be divided into three pathways. Mitochondria influence cell death through the intrinsic apoptotic pathway. When apoptosis is induced, mitochondrial membrane protein activation using the Bcl-2 family protein channels triggers mitochondrial outer membrane permeability and releases apoptosis proteins (such as Cyt c, Smac, etc.) into the cytoplasm. Cyt c and apoptotic protease activating factor 1 (APAF1) interact, forming apoptosomes and activating procaspase-9, which cracks caspase-3 and caspase-7, thus inducing cell apoptosis [49]. Many viruses promote viral spread by inducing cell death or maintaining persistent infection via inhibiting cell death. HCV inhibits cell apoptosis via disrupting mitochondrial dynamics. HCV infection induces the phosphorylation of DRP1Ser616, which triggers mitochondrial fission and mitophagy, thereby inhibiting cell apoptosis, which eventually promotes viral persistence [28]. CSFV infection is similar to HCV infection. CSFV and HCV infection trigger the occurrence of mitophagy via activating the PINK1 and Parkin pathways to clear impaired mitochondria and prevent the release of pro-apoptotic proteins, thereby inhibiting cell apoptosis and maintaining viral infection [28,31]. Drp1 silencing blocks mitochondrial fission, mitophagy, and up-regulated apoptosis signals induced by HCV and CSFV, reducing virion secretion [28,31]. Interestingly, HCV viral proteins play a different role in inducing apoptosis. For example, NS4A protein changes the intracellular distribution of mitochondria, causing mitochondrial damage and the release of Cyt c into the cytoplasm, thereby activating Caspase-3-mediated apoptosis [50]. E2 protein, transfected in Huh-7 cells, down-regulates Bcl-2 and up-regulates Bax, which may induce apoptosis through a mitochondrial-dependent caspase pathway [51]. Interaction of the core protein with 14-3-3ε protein releases Bax to activate apoptosis [52]. NS4B causes a decrease in mitochondrial membrane potential, activates caspase 9, and releases Cyt c, inducing apoptosis through the mitochondrial death pathway [53]. NS4A and NS3-4A proteins up-regulate Bax and translocate to the mitochondria, down-regulating the expression of anti-apoptotic protein Bcl-xL and activating caspase-9, thereby inducing mitochondrial-mediated death through the Bax and caspase cascade reaction, which eventually induces cell death [54]. Further research on the function and mechanism of viral proteins and the substances that inhibit the activity of viral proteins may provide new ideas for the treatment and drug development of chronic hepatitis.

HBV virus also induces apoptosis. HBx protein can strongly interact with p53 in the aggregated mitochondrial structure, leading to cell death [55]. Similarly, DENV induces p53-dependent mitochondria-mediated apoptosis [56]. By binding with Bax, HBx interferes with the interaction between Bax and 14-3-3epsilon, enhancing the transmigration of Bax to mitochondria, regulating the opening of mitochondrial permeability transition pores and releasing Caspase-3 and cytochrome C, and then mediating endogenous mitochondrial apoptosis [57,58]. HBV also inhibits apoptosis and maintains viral infection by changing mitochondrial dynamics. HBx can induce the ubiquitination of Mfn2, promote the expression of DRP1, lead to mitochondrial fission, and induce mitophagy through the PINK1-Parkin pathway to inhibit cell apoptosis and maintain cell survival and persistent infection of the virus [37]. In addition, SARS-CoV can also induce cell apoptosis. SARS-CoV 3a protein can activate caspase-9 and cytochrome c protein release from the mitochondria or activate caspase-8 through extrinsic signal(s) and cause Bid activation to modulate the mitochondrial death pathway [59]. SARS-CoV N protein induces the decrease of mitochondrial membrane potential and the increase of ROS and cytochrome C release, which mediate apoptosis [59,60]. In addition, SARS-CoV M protein induces mitochondrial cytochrome c protein release, which mediates cell apoptosis [61]. Similarly, SARS-CoV-2 3a protein may induce apoptosis [48].

Moreover, viruses can promote replication and spread by regulating cell death. For example, Rotavirus, a double-stranded RNA virus belonging to the Reoviridae family, can induce apoptosis. Recent studies have shown that NSP4 changes the mitochondrial membrane potential and mitochondrial permeability through interaction with the mitochondrial membrane protein adenine nucleotide translocator and voltage-dependent anion channel (VDAC), releasing cytochrome C, activating caspase, and up-regulating the apoptosis signal to mediate cell apoptosis [62]. In addition, Rotavirus infection can up-regulate the concentration of Bax and mediate apoptosis through the mitochondrial pathway [63]. On the other hand, in the early stage of Rotavirus infection, NSP1 inhibits cell apoptosis by activating the PI3K/Akt signaling pathway or inhibiting p53 regulation and ensuring early replication of the virus in the cell [64]. Rotavirus infection also mediates apoptosis by regulating mitochondrial dynamics. In the late stage of Rotavirus infection, NSP4 induces the Ser616 phosphorylation of Drp1 through CDK1 and participates in the recruitment of DRP1 to mitochondria, mediating mitochondrial fragmentation, releasing Cyt c, and activating caspase-9 and caspase-3 to induce apoptosis and facilitate the spread of the virus [65]. Similarly, influenza A virulence factor PB1-F2 targets the IMM and causes mitochondrial dysfunction and induces cell death through the endogenous mitochondrial pathway [38,66]. Zika virus is a single-stranded positive-stranded RNA virus belonging to the genus Flavivirus, and Zika virus infection can also reduce mitochondrial transmembrane potential, reduce the expression of Mfn2, and promote mitochondrial fragmentation, inducing cell apoptosis. Mitochondrial division inhibitor 1 (Mdivi-1), a small molecule inhibiting mitochondria fission, blocks mitochondrial fission and improves mitochondrial dynamics after Zika virus infection, thereby increasing cell survival [67].

Interestingly, various strategies to evade cellular immunity have evolved in viruses. For example, virus infection can induce cell apoptosis to facilitate shedding and hence dissemination. In addition, viruses can inhibit cell apoptosis through mitophagy, thereby ensuring their replication. At present, the mechanism between apoptosis and autophagy is not fully understood, but regulation of each process keeps cells in a balanced state [68,69]. Several studies have shown that many viruses can maintain viral infection by triggering mitophagy to prevent apoptosis. HCV clears fission mitochondria through mitophagy, thereby inhibiting cell apoptosis. Silencing DRP61 or Parkin can increase the secretion of cytochrome C, significantly increasing the apoptosis signaling and enhancing the activity of caspase3. These results suggest that HCV promotes viral persistence by attenuating apoptosis through mitophagy [28]. Porcine reproductive and respiratory syndrome (PRRSV), a single positive-strand RNA virus of the *Arteriviridae* family, can promote self-replication by disrupting mitochondrial dynamics, inducing mitophagy, and inhibiting cell apoptosis [70]. HBV induces mitochondrial fission and mitophagy molecules, which mediate mitochondrial fission and mitophagy, and reduces virus-induced cell apoptosis. Interfering with the production of mitophagy enhances the apoptosis signal and reduces virus replication [37]. Similarly, Newcastle disease virus (NDV), a single-stranded negative RNA virus that belongs to the *Paramyxoviridae* family, Porcine reproductive virus, and CSFV can inhibit cell apoptosis by inducing mitophagy, thereby promoting virus infection [31,71]. In the context of viral infection, how apoptosis regulates mitophagy and the molecular mechanism of the mutual regulation between apoptosis and mitophagy need further study.

**Figure 2:**
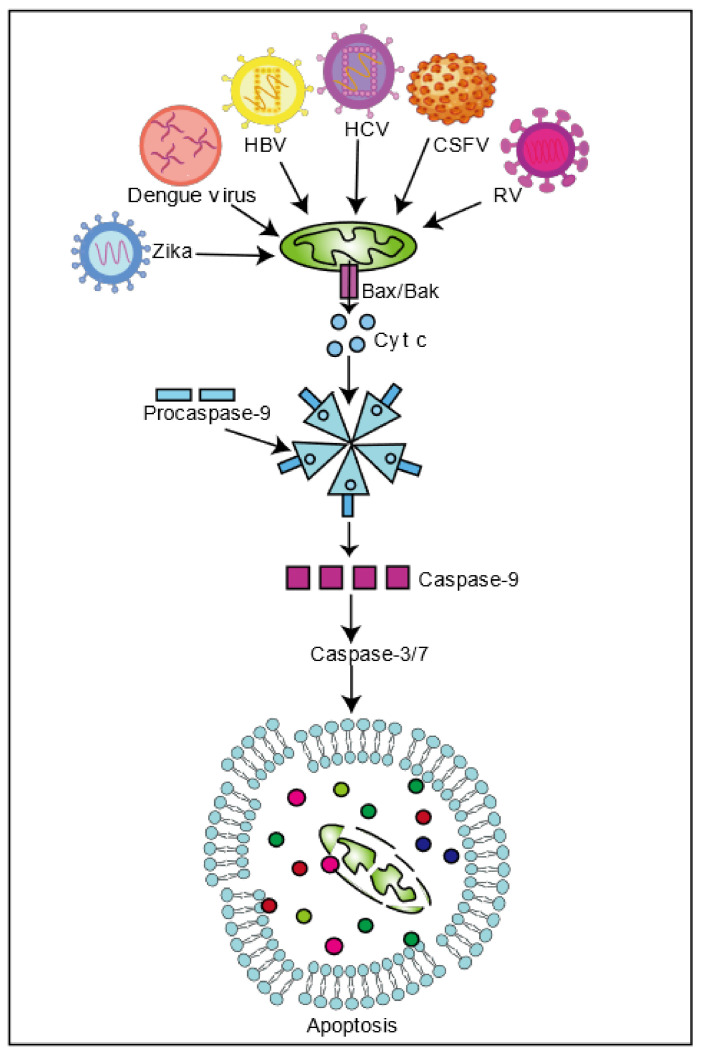
Viral infection regulates mitochondria-induced cell death. Different viruses mediate B-cell lymphoma 2 (Bcl-2) family proteins, release Cyt c, activate procaspase-9, and form apoptosomes, thereby inducing cell apoptosis.

In conclusion, viruses inducing apoptosis through the mitochondrial pathway maintains the niche of self-replication (Figure 2). Therefore, further research into the specific mechanism of virus-induced cell apoptosis will facilitate new antiviral drugs for different viruses.

## 4. Viral Infection Regulates Mitochondria-Induced Innate Immunity

When a virus infects cells, the host activates the innate immune system to recognize the virus through pathogen recognition receptors (PRRs), such as TLRs, RLRs, and NLRs. Here, we focus on viral infection regulating the mitochondria-mediated RLR signaling pathway. Many viral pathogen-associated molecular patterns (PAMPs) are recognized by the retinoic acid-inducible gene I (RIG-I) and melanoma differentiation-associated gene 5 (MDA5); RIG-1 and MDA5 undergo conformational changes that result in the exposure of the CARD domain to form a homologous oligomer. RIG-1 and MDA5 recognize and bind to each other through the N-terminal CARD domain and the N-terminal CARD domain of MAVS, forming MAVS prion-like polymers and activating downstream signaling pathways such as NF-κB and IRF3/7, thereby inducing the expression of inflammatory cytokines and interferons involved in the innate antiviral response. MAVS is located in the OMM as the pivotal adaptor protein of the RLR pathway. MAVS’ functions depend on its mitochondrial localization, confirming that mitochondria play an important role in the innate immune signaling pathway.

**Figure 3:**
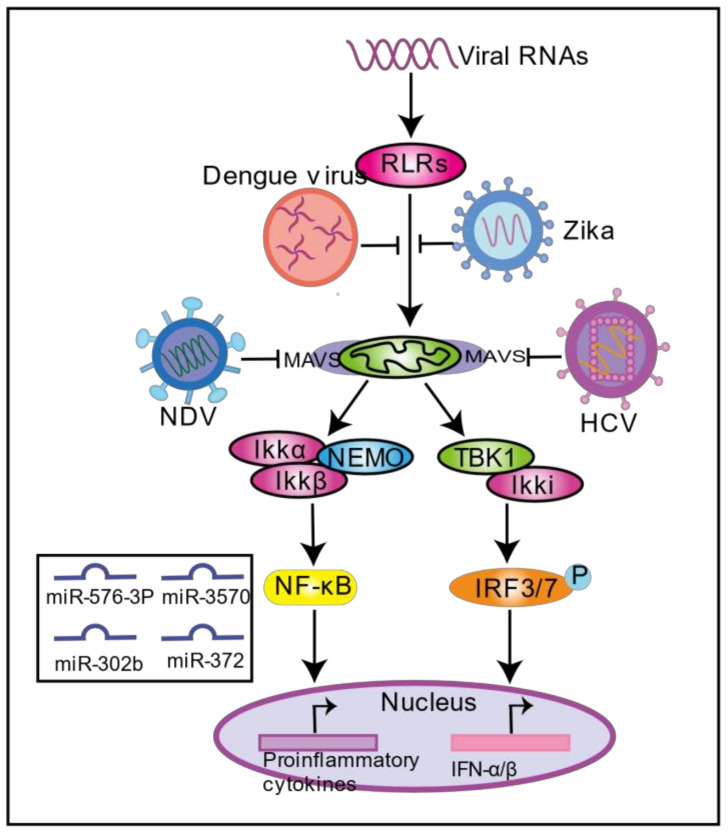
Viral infection regulates mitochondria-induced innate immunity. After the virus invades the cell, RLRs recognize the viral RNA and interact with the mitochondrial antiviral signal (MAVS) to activate the antiviral signal pathway. Different viruses evade host innate immunity by blocking the RLR signaling pathway.

More and more studies have shown that viruses developed a series of strategies to antagonize the RLR signaling pathway in mitochondria during the their evolution to escape from the host immune system (Figure 3). SARS-CoV 3b protein inhibits type I IFN production by blocking MAVS activity [72]. In addition, the Nsp13 protein and 9C protein of SARS-CoV-2 may be involved in the regulation of MAVS signal transduction, thereby mediating the innate immune response [48]. A recent study showed that SARS-CoV-2 infection in human colon epithelial cancer cells Caco-2 resulted in decreased expression of MAVS [73]. HCV can evade host immunity, causing chronic infection. NS3/4A can be localized in mitochondria and combined with MAVS. NS3/4A cleaves MAVS at Cys-508, causing the N-terminal fragment of MAVS to dislocate from the mitochondria and become an inactive fragment, which prevents the production of IFN [74,75]. Similarly, Bat Hepatovirus and Seneca Valley virus are RNA viruses, both belonging to the *Picornaviridae* family, that can also interfere with innate immune signal transduction by interacting with MAVs protein, thus maintaining viral infection [76,77]. Bat Hepatovirus 3ABC proteases interact with human MAVS and cleave MAVS at Glu^463^/Gly^464^ to inhibit the activation of IRF3 and NF-κB, thereby blocking the production of type I interferon in human cells [76]. Seneca Valley virus 3C protease depends on its protease activity to cleave MAVS at Q148, inhibiting the type I interferon [77]. In addition, the virus can also degrade MAVS through the proteasome pathway and block the RLR signaling pathway. For example, HBV HBx protein can interact with MAVS, promote the ubiquitination and degradation of MAVS, and inhibit the RIG-I-MDA5 pathway, which together reduce the production of IFN-β [78]. NDV V protein recruits E3 ubiquitin ligase RNF5 to mediate MAVS degradation through the proteasomal pathway to prevent IFN production [79]. Rotavirus VP3 protein targets mitochondria and mediates the phosphorylation of the SPLTSS motif in the proline-rich region of MAVS, causing MAVS to be degraded through the proteasome pathway, blocking the production of IFN-γ during Rotavirus infection of intestinal epithelial cells [80]. Viruses also inhibit the RLR signaling pathway by blocking the binding of MAVS to RIG-1 and MDA5. By binding to the 14-3-3-binding motif, Zika virus NS3 prevents RIG-1 and MDA5 from being transported to mitochondria, thereby blocking interferon production mediated by the RLR signaling pathway [81]. DENV NS4A binds to the N-terminal CARD-like (CL) domain and the C-terminal transmembrane (TM) domain of MAVS, which prevents MAVS from binding to RIG-I and inhibits the production of interferon [82]. Viruses can also evade host innate immunity by manipulating microRNAs, regulating a range of host immune systems through post-transcriptional regulation to block RLR signaling pathways. Vesicular Stomatitis Virus (VSV), a single-stranded negative RNA (ssRNA) virus of the family *Rhabviridae*, infection induces miR-576-3p through IRF3 and regulates MAVS and TRAF3 mRNAs to reduce type I expression interferon and avoid excessive inflammation [83]. Rhabdovirus infects miiuy croaker macrophages, inducing miR-3570 expression and targeting and suppressing MAVS expression, thus promoting the virus [84]. Studies have shown that miR-302b and miR-372 induced by viral infection may manipulate cell function and mitochondrial metabolism through aspartate glutamate transporter SLC25A12, thereby impairing MAVS-mediated innate immunity to antiviral viruses [85]. Interestingly, the introduction of mimics of miR-302b and miR-372 into cells can reduce NADH levels, resulting in an increase in the NAD/NADH ratio by up to 50%, a decrease in mitochondrial oxygen consumption, and ultimately a change in cellular metabolic pathways from the citric acid cycle to sugar digestion, while increasing the content of lactate [85]. The latest research shows that hepatitis B virus infection directly binds to MAVS through lactate dehydrogenase-dependent lactic acid to prevent MAVS from mitochondrial aggregation and localization, thereby blocking the RLR signaling pathway [86]. Since lactic acid plays a negative regulatory role in calf-mediated innate immune response [87], these two miRNAs may affect innate immunity through lactic acid regulation.

## 5. Viral Infection Regulates Mitochondrial Metabolism

Mitochondria are the energy metabolism centers of cells; they produce ATP by regulating macromolecular metabolism of carbohydrates, amino acids, and fatty acids. The main energy source of the cell is dephosphorylated by the ATP molecule into an adenosine diphosphate (ADP) molecule. For this process to continue, cells need to break down some macromolecular metabolites through pathways such as glycolysis, the tricarboxylic acid cycle, and oxidative phosphorylation. Glucose is the primary source of energy for cells. In the cytoplasm, two molecules of ATP are produced by glycolysis from one molecule of glucose, generating two pyruvate molecules. To optimize ATP production, cells undergo oxidative phosphorylation (OXPHOS), which oxidizes pyruvate into the mitochondrial matrix through the mitochondrial pyruvate carrier (MPC) with the tricarboxylic acid cycle. Finally, complete oxidation of one glucose molecule through the mitochondrial electron transport chain generates 36 ATP molecules. Although oxidative phosphorylation produces high energy efficiency, it is a slow process and cannot meet the energy requirements of rapidly dividing cells, such as activated immune cells or cancer cells. Therefore, these cells need to initiate aerobic glycolysis (also called the Warburg effect) to produce energy quickly to maintain their activity. In addition, during starvation and emergency, lipase degrades lipids into free fatty acids, which enter mitochondria for fatty acid β oxidation, thus maintaining the balance of cellular energy metabolism.

Many viruses can actively reshape host cell metabolism to enhance intracellular survival. HCV infection causes changes in cell metabolism, which increases the carbohydrate flux during glycolysis and decreases the activities of aerobic oxidative phosphorylation and the citric acid cycle, which may direct the cell towards the Warburg Effect quite quickly, within a few days or weeks after infection of a cell [88,89,90]. In a recent study, some critical components of the mitochondrial respiratory chain complex were found to be down-regulated six days after HCV infection, including MT-ND1, MT-ND3, MT-ND4, MT-ND4L, and MT-CO2 [91]. In addition, the down-regulation of MTND, COX, and F_0_/F_1_ATP synthase was found in the HCV-infected CD8^+^T cell cycle [92,93]. It has been shown that HCV systematically limits the activity of oxidative phosphorylation by altering the expression of the mitochondrial respiratory chain complex [94]. HIF-1α and the proto-oncogene c-myc are significantly expressed in HCV-infected cells, inducing the expression of several glycolytic key enzymes, including glucokinase (G.K.), phosphoglucose-1 (PFK-1), and pyruvate kinase (P.K.) [95,96,97]. In addition, HCV infection induces upregulation of Hexokinase 2 expression and enhances Hexokinase activity through interaction with HCV protein NS5a [98]. DENV infection also induces the upregulation of glucose transporter 1 and hexokinase 2 [99]. Inhibition of the glycolytic pathway significantly reduced the RNA synthesis of DENV and the production of infectious virions, revealing that DENV can remodel cellular glycolysis to maintain its replication [99]. Interestingly, DENV proteins have different effects on host metabolism. DENV NS1 protein interacts with GAPDH to enhance the glycolytic activity of GADPH [100]. However, the interaction of DENV NS3 protein with GAPDH resulted in reduced GAPDH glycolysis activity [101]. HCV and DENV infection can reshape cell metabolism, enhance mitochondrial fatty acid oxidation, and provide energy [102,103,104]. Meanwhile, inhibition of fatty acid transport to mitochondria and regulation of β-oxidation can affect viral replication [103]. The Zika virus can utilize host resources and reprogram cell metabolism in different cells to regulate the cell’s state in different metabolic pathways, thus facilitating its self-replication [105,106,107,108]. HIV replicates in CD4^+^ T cells and leads to metabolic reprogramming from oxidative phosphorylation to aerobic glycolysis [109]. HIV infection induces the increase of glucose transporter-1, the uptake of more glucose, and the up-regulation of glycolytic enzymes lactate dehydrogenase A (LDHA) hexokinase-1, thus activating aerobic glycolysis, which is conducive to HIV reverse transcription, integration, and virion production [110,111,112,113]. In addition to increasing aerobic glycolysis, HIV-infected CD4^+^ T cells can cause glutamine metabolism and reuse glutamine during productive HIV infection [114,115]. In addition to glucose and glutamine as the primary energy sources, HIV also uses fatty acid oxidation as an energy source to infect CD4^+^ T cells [115]. A recent study showed that HIV infection induces aerobic glycolysis, which helps control the quality of the virus by controlling the factors packaged into the particles to maintain infectivity [116]. Although mitochondrial metabolism is closely related to viral infection, the mechanism by which viruses target mitochondrial metabolism and how viruses utilize the energy produced by cell metabolism is still unclear.

## 6. Concluding Remarks

Over the past few decades, mitochondria have been shown to play an important role in viral infection and host innate immunity; however, the role of mitochondria in the host–virus interaction needs to be further studied. Viral infections can create a viable ecological niche for themselves by manipulating mitochondrial function. The virus induces mitochondria-induced cell death and the mitochondria-mediated innate immune system to facilitate its replication and transmission by regulating mitochondrial dynamics. In recent years, the role of mitochondria as the regulatory center of cell metabolism has attracted more attention. Viruses can manipulate cell metabolism, reprogram metabolic pathways, and reuse metabolites to maintain viral niches in cells. However, research on the mitochondria and its metabolism is still in its infancy. Delving into the mechanisms by which viruses use mitochondria-mediated cell metabolism to maintain infection is an exciting area for future research.

## Data Availability

Not applicable.
